# Healthcare-Associated SARS-CoV-2 Transmission—Experiences from a German University Hospital

**DOI:** 10.3390/microorganisms8091378

**Published:** 2020-09-08

**Authors:** Carlos L. Correa-Martínez, Vera Schwierzeck, Alexander Mellmann, Marc Hennies, Stefanie Kampmeier

**Affiliations:** 1 Institute of Hygiene, University Hospital Münster, 48149 Münster, Germany; Carlos.Correa@ukmuenster.de (C.L.C.-M.); Vera.Schwierzeck@ukmuenster.de (V.S.); Alexander.Mellmann@ukmuenster.de (A.M.); 2 Institute of Virology, University Hospital Münster, 48149 Münster, Germany; Marctim.Hennies@ukmuenster.de

**Keywords:** SARS-CoV-2, COVID-19, healthcare-associated transmission, health care workers, C_t_-value, attack rate

## Abstract

During the current severe acute respiratory syndrome coronavirus 2 (SARS-CoV-2) pandemic, healthcare systems worldwide have to prevent nosocomial SARS-CoV-2 transmission while maintaining duty of care. In our study, we characterize the transmission dynamic of SARS-CoV-2 in inpatients and healthcare workers (HCWs) at the University Hospital Münster (UHM) in northwest Germany. We identified 27 cases of healthcare-associated SARS-CoV-2 infections (4 inpatients and 23 HCWs) who had contact with patients and/or HCWs without the use of adequate PPE. The contacts of these index cases were followed up for SARS-CoV-2 infection after unprotected exposure and a quantitative measure of probability of becoming infected, the attack rate, was calculated. In addition, transmission was evaluated in the context of infection control measures established during the pandemic and we compared the epidemiological data of all index cases, including symptoms and C_t_ values of virology test results. The overall attack rate in the hospital setting was 1.3% (inpatients 0.9%, HCWs 1.6%). However, during an outbreak, the attack rate was 25.5% (inpatients 20.0%, HCWs 29.6%). For both scenarios, HCWs had a higher attack rate illustrating their role in healthcare-associated SARS-CoV-2 transmission. Taken together, our experiences demonstrate how infection control measures can minimize the transmission of SARS-CoV-2 in the healthcare setting.

## 1. Introduction

The ongoing severe acute respiratory syndrome coronavirus 2 (SARS-CoV-2) pandemic constitutes an unprecedented sanitary emergency that continues to pose a major challenge to healthcare systems worldwide. As of late July 2020, seven months after the emergence of the first cases of coronavirus disease 2019 (COVID-19) in the city of Wuhan, People’s Republic of China, approximately 600,000 deaths have occurred due to COVID-19 worldwide, with over 14 million cases reported in 219 countries and territories [1]. In Germany, over 200,000 cases have been reported so far [2]. This has led to the update and implementation of the National Pandemic Plan, a series of infection prevention and control strategies that have been adapted and applied by each Federal State according to local conditions [3,4].

In spite of initial controversy, current evidence indicates that person-to-person transmission of SARS-CoV-2 occurs through contact (direct and indirect), droplets, and (indoor) aerosols [5]. In addition, the newest reports describe a previously unknown transplacental transmission [6]. These multiple mechanisms, along with an infectious dose estimated to be similar or lower to that of SARS-CoV-1 and the possibility of infectivity within the incubation period, show that SARS-CoV-2 has a higher spread potential [7,8]. Considering the current lack of vaccines and antiviral drugs for causal treatment, infection prevention and control strategies represent the cornerstone of health interventions aimed at tackling the pandemic [5].

It is widely known that the healthcare setting offers optimal conditions for the spread of viral agents. Several factors contribute to this, such as the close interaction between patients and healthcare workers (HCWs), as well as the increased susceptibility of immunocompromised patients and the nature of certain medical procedures that may enhance the risk of exposure [9,10]. 

Reports which originated from previous coronavirus epidemics in recent decades offer valuable information for estimating the potential nosocomial spread of SARS-CoV-2. Evidence from the severe acute respiratory syndrome (SARS) and middle east respiratory syndrome (MERS) epidemics indicates that, while these viral agents may differ in their exposure and transmission patterns, large nosocomial outbreaks of both agents were usually preceded by super-spreading events caused by the delayed diagnosis of index cases [11]. During the 2002/2003 SARS pandemic, a highly efficient transmission in the hospital setting was documented for this agent, with nosocomial spreading events being identified as responsible for the amplification and propagation of the pandemic [12]. Furthermore, unrecognized cases of patients displaying few or unspecific symptoms triggered nosocomial outbreaks in several countries [12]. Similarly, unsuspected infections represent a significant challenge in the current SARS-CoV-2 pandemic, as indicated by reports of nosocomial spread originated from cases not identified upon admission [13,14] or undiagnosed HCWs [15]. Moreover, infectivity of patients within the incubation period further hampers appropriate contact tracing and prompt implementation of control measures [14,16,17]. The present study sheds light on the nosocomial transmission of SARS-CoV-2 in the context of a large university hospital in Germany, focusing on the role of undetected cases among patients and HCWs. Moreover, the study aims to identify strategies for effective resource management and infection control measures useful to minimize nosocomial transmission at different stages of the pandemic. 

## 2. Materials and Methods

### 2.1. Hospital Characteristics and Infection Control Measures

The University Hospital Münster (UHM) is a tertiary care center with 1500 beds (80 in intensive care units, 43 in intermediate care units, IMC). The UHM admits 62,000 patients yearly, a number which has decreased by approximately 40% during the COVID-19 pandemic. It is located in the Federal State of North-Rhine Westphalia (NRW), in the northwest of Germany. During the first wave of the pandemic in mid-March, SARS-CoV-2 incidence peaked at 5.8 and 6.0 cases per 100,000 inhabitants in NRW and Germany, respectively. Following the nationwide lockdown measures, these values decreased to 0.1 and 0.2 cases per 100,000 inhabitants in May, respectively [2].

In response to growing infection cases, several measures were adopted at our hospital. Tests were carried out following the national case definition and testing strategy centrally defined by the German Public Health Authority [18,19]. These recommendations were implemented accordingly at different stages of the pandemic.

Further measures and protocols were established internally at the UHM. In particular, the emergency department was forced to adopt processes for COVID-19 patients quickly. For this reason, new isolation capacities, the use of shipping containers for outpatient testing and a new COVID-19 ward including 32 IMC isolation rooms were established [20]. Furthermore, occupation in normal wards was limited to two patients per room, visitor access was restricted to a minimum (e.g., relatives of terminal patients) and working trips of all employees outside of the city were forbidden. Enhanced infection control measures were planned for confirmed and suspect cases, including isolation in COVID-19 wards and use of personal protective equipment (gloves, gowns, FFP2 masks and safety goggles).

**Figure 1:**
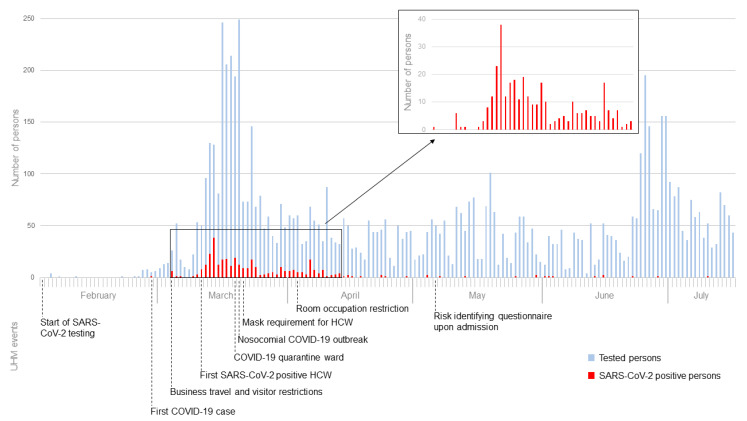
Management of the COVID-19 pandemic in the University Hospital Münster (UHM). Numbers of persons tested and with SARS-CoV-2 positive test results are listed in an epidemiologic curve. Events and prevention strategies in the UHM are mentioned in the lower part.

SARS-CoV-2 testing was started in the beginning of February at the UHM, receiving the first positive test result in the end of February. On March 20th the first nosocomial outbreak took place in the UHM in a pediatric dialysis unit [15]. Thereafter, wearing surgical masks was obligatory for every HCW in the UHM. After the first wave of infections, in the beginning of May, a questionnaire identifying potential COVID-19 symptoms and risk factors was introduced to detect potentially infected inpatients upon admission (for exact timeline see also Figure 1).

In the context of this study, index persons were defined as inpatients or HCWs tested positively for SARS-CoV-2 and having contact with patients or HCWs without adequate contact precautions. Contact persons were defined as persons having contact with SARS-CoV-2 positive HCWs or patients within 48 h prior to the positive test result and without personal protection. Protection was assumed if personal protective equipment (including FFP2 masks) was worn during contact with the infected person. Contact persons were defined infected, if SARS-CoV-2 was detected within 14 days after unprotected contact via nasopharyngeal swab sample.

### 2.2. SARS-CoV-2 Testing

Testing was performed immediately following clinical diagnosis, with results being available within 24 h. SARS-CoV-2 was detected employing the Rotor-Gene Q platform (QIAGEN N.V., Venlo, Netherlands), targeting two separate genes via real-time RT-PCR as described previously [21]. Detection of envelope (E) gene was used as a screening test and detection of RNA-dependent RNA polymerase (RdRp) gene was used for confirmation. The threshold cycle value (C_t_-value), which is inversely proportional to the viral load, was documented for every SARS-CoV-2 positive index patient.

### 2.3. Epidemiologic Investigation and Contact Tracing

Epidemiological data of suspect cases were collected upon testing, including information on symptoms, travel history and contact to confirmed cases or infection clusters. Contact tracing was carried out using data centrally stored in the hospital’s patient management software and complemented with phone interviews when appropriate.

### 2.4. Statistical Analysis and Calculation of Attack Rate

Data are given as absolute numbers or percentages, if not stated otherwise. Attack rates, an indication of the proportion of subjects becoming infected among a group exposed to an index case [22] were calculated by dividing the number of exposed infected by the number of total exposed persons, in both outbreak settings only (= outbreak attack rate) and overall persons (= overall attack rate) using Microsoft Excel^®^.

## 3. Results

From February to July 2020, 7760 HCWs, inpatients and outpatients were tested for SARS-CoV-2, of which in total 357 persons were positive (Figure 1). According to the above-mentioned criteria, 27 index cases of hospital-associated SARS-CoV-2 infection could be identified and were followed up to investigate any further transmission in the hospital. Of these cases, 23 were HCWs and four were incidentally detected inpatients, who were asymptomatic on admission. Clinical characteristics of these index persons while tested are summarized in Table 1. In total, 913 contact cases could be identified (428 patients and 485 HCWs), of whom in total four patients and eight HCWs were infected, respectively, during a nosocomial outbreak on a pediatric hemodialysis unit in early March. All 23 infected HCWs were put under home quarantine and underwent two SARS-CoV-2 tests before resuming work. Infected patients were isolated following infection control measures recommended by German Public Health authority [23]. Subsequently, no additional healthcare-associated infection or positive SARS-CoV-2 test in a contact person was detected. C_t_-values of index persons ranged from 17.96 to 41.07 (Table 1). The total outbreak attack rate was 25.5% (20.0% for patients and 29.6% for HCWs), while the overall attack rate was only 1.3% (0.9% for patients and 1.6% for HCWs).

## 4. Discussion

During the current COVID-19 pandemic, preventing nosocomial SARS-CoV-2 transmission is one of the major challenges for hospitals. From an infection control point of view, two entities of SARS-CoV-2 positive individuals pose the greatest risk as source of transmission in the healthcare setting: SARS-CoV-2 positive patients before diagnosis and SARS-CoV-2 positive HCWs who are not yet aware of their infection.

While the overall attack rate of SARS-CoV-2 was higher for HCWs, HCWs represented the majority of our index cases. As part of our study, we reviewed the case history of SARS-CoV-2 positive HCWs. Transmission outside the hospital setting was the main source of infection for HCWs. This could be explained by the prevailing epidemiological conditions in the region, with the Federal State of North-Rhine Westphalia, in which the UHM is located, being one of the most affected by the pandemic in Germany [2]. In view of this challenging epidemiological context, the prompt establishment and implementation of occupational health policies aimed at the early detection and control of cases was shown to be instrumental in identifying infected hospital staff thus preventing further spread.

Evidence regarding attack rate of SARS-CoV-2 in the hospital setting is scarce. However, the attack rate in our hospital was low (1%) and below the values reported in several transmission events, ranging from 4 to 100% [24,25,26,27]. By early March, the UHM had established several infection control measures that might have contributed towards this result, including the use of facemasks in common areas, reduction in bed occupancy, and restriction of the number of visitors. Interestingly, no nosocomial outbreak occurred since facemasks were mandatory at the UHM. In addition, each patient admitted to the UHM was assessed for SARS-CoV-2-associated symptoms or contact to SARS-CoV-2 infected individuals with the help of a standardized questionnaire. This allowed for the rapid identification of any history of contact to positive cases or symptoms suggestive of SARS-CoV-2, enabling the implementation of adequate contact precautions upon admission. 

Although the C_t_ values of two index cases were identical, suggesting similar viral loads, only one index case caused a nosocomial outbreak. This observation highlights the complexity of superspreading events. This concept was previously applied in the context of the 2003 SARS epidemic in order to describe the infection of numerous persons by one single infected patient. The occurrence of such events in healthcare centers led to the revision and strict implementation of infection control measures in order to contain the spread of SARS-CoV-1 [28,29]. While a high viral load is one contributing factor, there are also behavioral and environmental factors, such as social contacts of the infected individual.

In total, 23 of 27 SARS-CoV-2 index persons were HCWs, indicating their role as important link in the introduction of this pathogen from the community into the enclosed hospital setting. The attack rate was higher among HCWs than among patients, while the C_t_-value, an indirect indicator for disease progression [30], remained similar in both groups. This could be due to higher mobility of HCWs as compared to patients. The close interaction between HCWs during shifts could also be of relevance, especially in situations in which keeping the recommended minimum safe distance is not possible due to structural or logistical limitations. Additional HCW interaction outside the hospital should also be taken into consideration in explaining this phenomenon. 

There are some limitations regarding the present study. Our sample size is relatively small and comprises only monocentric data. Differences in the C_t_ values and attack rates between both HCWs and patients might be detected by analyzing larger samples from different hospitals. Furthermore, although person-to-person contact is the best described mechanism of transmission, exposed persons may become infected through alternate modes such as contact with contaminated environments and indoor air contamination via aerosols. This interesting question should be addressed in future studies to identify the contribution of different transmission modes to the overall attack rate of SARS-CoV-2 in the hospital setting. Variation in host susceptibility results might also influence infection rates and disease progression, but was not determined here due to the epidemiological nature of the study.

## 5. Conclusions

Undiagnosed HCWs are key in the importation of SARS-CoV-2 into the hospital setting, and show a greater spread potential than patients at similar levels of infectivity. The introduction of unsuspected infected patients and HCWs cannot be fully avoided in the context of a pandemic. In our experience, further transmission within the hospital can be significantly minimized by implementing anamnestic assessment of patients upon admission as well as timely reporting, testing and quarantine for both patients and HCWs.

## Figures and Tables

**Table 1:** Characteristics of index persons (HCW and patients) upon diagnosis.

Index Person no.	Date of Diagnosis	Age	Gender	Symptoms									C_t_-Value
				Fever	Cough	Sore Throat	Rhinorrhea	Loss of Taste/Smell	Muscle Ache	Dyspnea	Diarrhea	Headache	
1	16/03/20	27	F	+	-	-	-	-	-	-	-	-	30.97
2	17/03/20	45	F	+	+	+	-	-	+	-	-	+	22.56
3	17/03/20	37	F	-	+	+	-	-	-	-	-	-	35.24
4	17/03/20	34	F	-	-	-	-	-	-	-	-	-	24.56
5	18/03/20	40	M	-	+	-	-	-	-	-	-	-	38.04
6	19/03/20	46	M	-	+	+	+	-	-	-	-	-	21.92
7	19/03/20	36	M	-	-	-	-	-	-	-	-	-	21.08
8	19/03/20	31	F	-	-	-	-	-	-	-	-	+	27.35
9	19/03/20	62	F	-	+	+	-	-	-	-	-	+	33.18
10	19/03/20	28	F	-	+	+	+	-	-	-	-	+	24.12
11	19/03/20	26	M	-	+	-	-	-	-	-	-	+	18.53
12	19/03/20	27	M	-	-	-	-	-	-	-	-	-	27.64
13	19/03/20	66	M	+	+	-	-	-	+	+	-	-	22.87
14	20/03/20	55	F	+	-	-	-	-	-	-	-	+	17.96
15	20/03/20	26	M	+	+	-	-	-	+	-	-	-	18.00
16	23/03/20	28	F	+	+	-	-	-	-	+	-	+	22.91
17	23/03/20	29	M	+	+	-	+	-	-	-	-	-	19.86
18	23/03/20	52	F	-	+	-	+	+	-	-	-	+	27.20
19	23/03/20	19	F	+	+	+	+	-	-	-	-	-	17.96
20	24/03/20	79	F	-	+	-	+	-	-	-	-	+	27.95
21	01/04/20	88	M	-	-	-	-	-	-	-	-	-	32.40
22	06/04/20	23	F	-	-	-	-	-	-	-	-	-	39.46
23	08/04/20	24	M	-	+	+	-	-	-	+	+	-	35.02
24	09/04/20	52	F	-	-	-	-	-	-	-	-	-	40.46
25	09/04/20	64	M	-	-	-	-	-	-	+	-	-	22.86
26	12/04/20	33	F	-	-	-	-	-	-	-	-	-	41.07
27	17/05/20	18	F	-	-	-	-	-	-	-	-	-	ND
Average/ Quantity		42	-	8	15	7	6	1	3	4	1	9	27.35

ND—not determined; HCW—healthcare workers.
